# Pydpiper: a flexible toolkit for constructing novel registration pipelines

**DOI:** 10.3389/fninf.2014.00067

**Published:** 2014-07-30

**Authors:** Miriam Friedel, Matthijs C. van Eede, Jon Pipitone, M. Mallar Chakravarty, Jason P. Lerch

**Affiliations:** ^1^Mouse Imaging Centre, Hospital for Sick ChildrenToronto, ON, Canada; ^2^Kimel Family Translational Imaging-Genetics Research Laboratory, Research Imaging Centre, Centre for Addiction and Mental HealthToronto, ON, Canada; ^3^Department of Psychiatry, Institute of Biomaterials and Biomedical Engineering, University of TorontoToronto, ON, Canada; ^4^Rotman Research InstituteToronto, ON, Canada; ^5^Department of Medical Biophysics, University of TorontoToronto, ON, Canada

**Keywords:** neuroimaging, pipeline, image registration, software, Python

## Abstract

Using neuroimaging technologies to elucidate the relationship between genotype and phenotype and brain and behavior will be a key contribution to biomedical research in the twenty-first century. Among the many methods for analyzing neuroimaging data, image registration deserves particular attention due to its wide range of applications. Finding strategies to register together many images and analyze the differences between them can be a challenge, particularly given that different experimental designs require different registration strategies. Moreover, writing software that can handle different types of image registration pipelines in a flexible, reusable and extensible way can be challenging. In response to this challenge, we have created Pydpiper, a neuroimaging registration toolkit written in Python. Pydpiper is an open-source, freely available software package that provides multiple modules for various image registration applications. Pydpiper offers five key innovations. Specifically: (1) a robust file handling class that allows access to outputs from all stages of registration at any point in the pipeline; (2) the ability of the framework to eliminate duplicate stages; (3) reusable, easy to subclass modules; (4) a development toolkit written for non-developers; (5) four complete applications that run complex image registration pipelines “out-of-the-box.” In this paper, we will discuss both the general Pydpiper framework and the various ways in which component modules can be pieced together to easily create new registration pipelines. This will include a discussion of the core principles motivating code development and a comparison of Pydpiper with other available toolkits. We also provide a comprehensive, line-by-line example to orient users with limited programming knowledge and highlight some of the most useful features of Pydpiper. In addition, we will present the four current applications of the code.

## 1. Introduction

Understanding the relationship between genotype and phenotype and brain and behavior is a core biomedical research challenge in the twenty-first century (Henkelman, 2010; Paus, 2010). Key recent developments have relied on three-dimensional neuroimaging in humans and animal models to aid in this endeavor. Part of the challenge of using neuroimaging to provide insight into neuroscience questions is quantitatively assessing large amounts of data in an automated, accurate and high throughput manner. Typically, a single study will produce anywhere from twenty to hundreds of images, where the end goal is the assessment of differences in neuroanatomy due to factors such as genotype, behavioral training, environment and disease.

Multipe algorithms have been developed for the analysis of neuroimaging data, ranging from tissue classification (Zijdenbos et al., 2002) to computational geometry (Fischl and Dale, 2000; Macdonald, 2000; Kim et al., 2005) to image registration and automatic segmentation (Collins et al., 1995; Heckemann et al., 2006; Chakravarty et al., 2013) or combinations thereof (Ashburner and Friston, 2000; Good et al., 2001). Image registration in particular will be the primary focus of this work, given its wide range of applications in humans (Gogtay et al., 2004; Joshi et al., 2007, 2012; Hyde et al., 2009; Klein et al., 2009; Durrleman et al., 2013) and animal models (Spring et al., 2007; Lau et al., 2008; Lerch et al., 2008; Maheswaran et al., 2009; Ellegood et al., 2013). Image registration determines the transformation mapping one image into the space of another, where the difference between these two images is thus encoded in that transformation. The analysis of those transformations, termed alternately Deformation Based Morphometry (DBM) or Tensor Based Morphometry (TBM), then produces global and local measures of changes in volume, position, and shape (Chung et al., 2001; Lepore et al., 2006).

Given that neuroimaging studies consist of more than just two images, strategies are needed to analyze entire datasets to identify shape or volume differences and provide a common space for performing analyses. There are a number of such image registration paradigms currently in use. One common approach is to align all images in a study to a common coordinate system, such as Talairach or MNI space (Evans et al., 2012). Alternatively, additional power to identify shape differences can be gained when all subjects in a study are aligned toward a single template that is representative of the population being studied (Mazziotta et al., 2001; Fonov et al., 2011). In the event that such a template does not exist, a study-specific template can be created from all subjects in the study (Guimond et al., 2000). One way to do this is through iterative, group-wise registration. In this procedure, all scans are aligned to a common target, then resampled with the resulting transforms into the target space. These resampled images are then averaged, creating a target for a subsequent alignment (Kovačević et al., 2005). The final average is then used as common space from which to analyze shape differences in the population.

**Figure 1:**
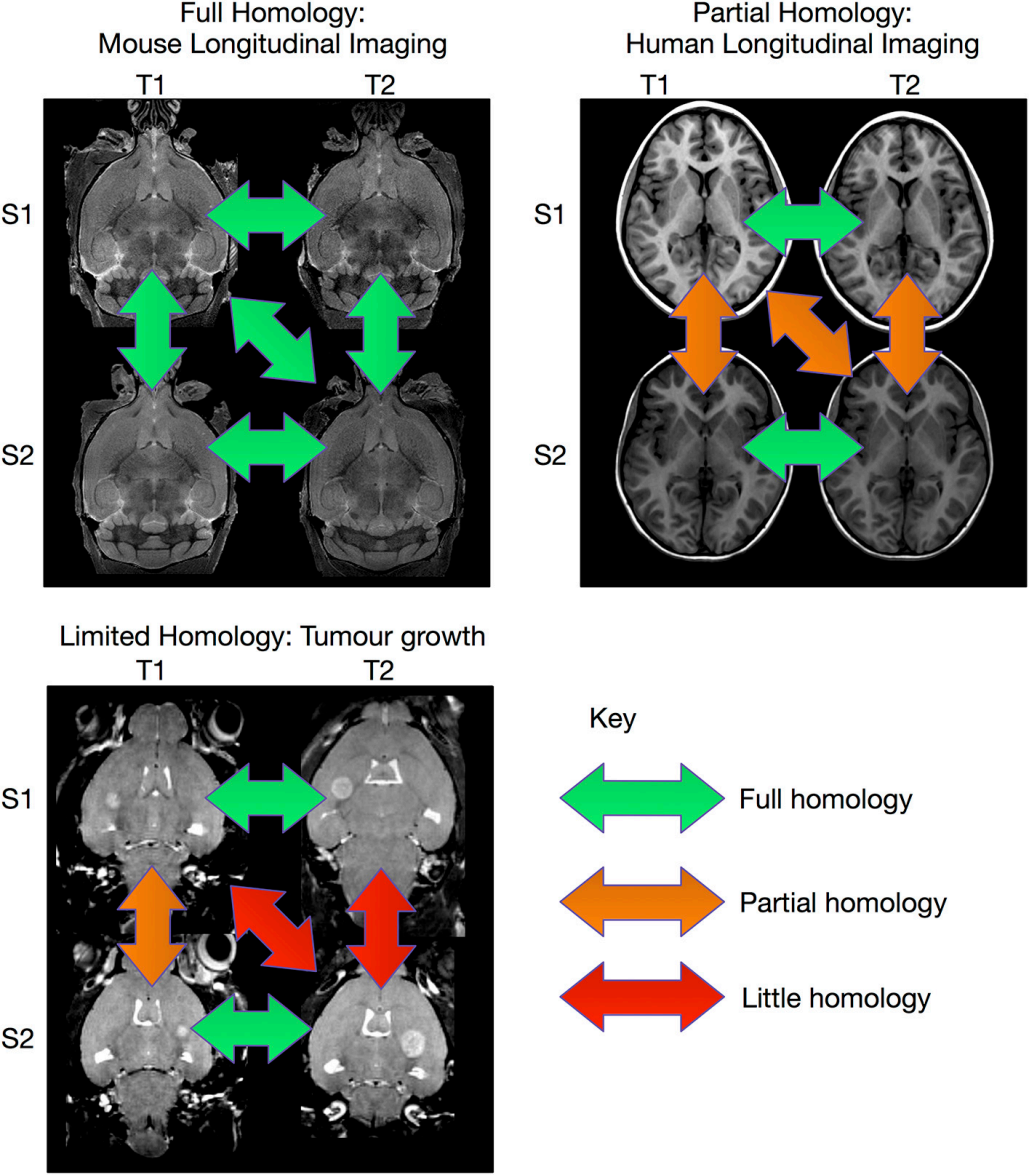
**Overview of registration scenarios**. In the case of aligning cross-sectional adult mouse brains full homology exists between any pair of brains. Human longitudinal data, on the other hand, has full homology between scans of the same subject but more limited homology between different subjects. In the case of pathology, such as brain tumor growth, homology can only be found with a sufficiently sampled time-series, but is lost due to idiosyncratic tumor growth across subjects. (Tumor data courtesy Lisa Gazdzinski and Brian Nieman, The Hospital for Sick Children; see Gazdzinski and Nieman, 2014).

The image registration processes described above are extremely effective when sufficient homology between all subjects in the study exist so that they can be registered to a common coordinate system. However, there are experiments where this is not possible (see Figure 1). This can be particularly true for longitudinal studies, where the same subject is scanned at multiple time points. In the case of early brain growth (Studholme, 2011; Szulc et al., 2013) or the growth of a tumor (Gazdzinski and Nieman, 2014), the anatomy of the brain changes to such an extent that insufficient homology exists to accurately register early time points to late ones. In spite of these difficulties, it is often possible to accurately register adjacent time points together if the time-series was densely sampled (Lerch et al., Manuscript in preparation). The resulting transforms can be concatenated and used to calculate shape changes from a common coordinate space.

A hybrid of the two registration paradigms mentioned above can provide additional power to detect shape differences. Here longitudinally acquired scans from the same subject are aligned to each other, a per subject average image generated from those registrations, and these average images are then aligned across all subjects. This process allows for high fidelity registrations within subjects; after early brain development is complete and absent severe disease processes, homology across time within subjects is much higher than homology across subjects. This is particularly true with regards to ideosyncratic cortical folding patterns (Mangin et al., 2010). The second step of registering the per subject average images together then provides a common coordinate space so that the longitudinal data can be analyzed across the study population and, in so far as homology exists, shape differences across subjects computed.

Underlying the different types of image registration described above are many common features. The most obvious of these is the ability to align two brains to a common space, often in a multi-step procedure, and subsequently make use of the resulting output transform in a meaningful way. These transforms must be concatenated appropriately so that deformation fields can be calculated, regardless of the type of registration or common space. Moreover, as part of the registration process, brains must be resampled, derivatives calculated from the transforms, and segmented atlases brought into the common space of each study, to cite a few examples. Finally, in order for a registration to be successful, an underlying framework must be present to run each command in the appropriate order, keep track of dependencies (e.g., transforms must exist before they can be concatenated), output useful log files in case debugging is needed, and save the necessary files for statistical analysis in an organized fashion.

In this paper, we present Pydpiper: the computational framework we have developed to address these registration challenges. We wrote this toolkit with the following principles as paramount: (1) high-level coding should be as simple as possible for those with less coding experience (advanced users can still easily get “under-the-hood” to create new modules); (2) individual building blocks of code should be as modular as possible, easy to subclass, and geared toward a range of biologically relevant applications; (3) complete, runnable pipelines containing thousands of stages and addressing the registration scenarios described above should be available “out-of-the-box”; (4) at the end of any pipeline, there should be an option to calculate the derived volumes necessary for TBM based statistics, using a module that contains all of the required stages; (5) we should include a robust file handling class to keep track of naming schemes and file interactions across many modules in a single application. This class not only simplifies coding, but also allows seamless access to files created at any point in the pipeline. These principles influenced design choices all the way through our code hierarchy, including mechanisms of creating and combining pipelines as well as providing high level access to multiple image registration routines.

The rest of this paper is structured as follows. First, we will discuss existing neuroimaging software toolkits and describe and how Pydpiper fits into this space. Then, we will describe the underlying, application-independent pipelining framework that comprises Pydpiper; next, we will discuss the main levels of Pydpiper class structure, and how different classes may be pieced together to create new classes and applications; finally, we will describe in more detail the applications we have written to address four different registration challenges. To augment these sections, we include a worked example in section 5 that compares a registration pipeline written in Pydpiper with the corresponding code as it would be run manually on the command line. Finally, we conclude by highlighting the innovations Pydpiper brings to the existing space of pipelining frameworks used to solve neuroimaging problems.

## 2. Motivation and existing solutions

As described in the previous section, there are a number of commonalities that underlie seemingly disparate image registration strategies, all of which are frequently used in our group, and we wanted a toolkit to address all of them in a seamless way, focusing on the four core design principles listed above. Moreover, we found ourselves in a position that is common among many labs: frequently, a single executable and its related functions and libraries are coded to run one type of registration protocol and are not easily adaptable to other applications. In our case, we have used a highly successful pipeline environment, MICe-build-model (see https://wiki.mouseimaging.ca/display/MICePub/MICe-build-model), to do iterative, group-wise registration (Lerch et al., 2011), described both above and more fully in section 4.2. Unfortunately, using this tool to create any of the other types of pipelines was cumbersome, time-consuming and in many instances, was not fully-automated or required too many manual, intermediate steps. What's more, modification of code like this (whether written by us or others) can be prohibitively time consuming for neuroimaging students and post-docs who do not have an extensive computer science background. Finally, in our work on registration sensitivity (van Eede et al., 2013), we developed a set of optimized registration parameters for our iterative group-wise registration procedure. We wanted to adapt these and flexibly share them among different registration modules, but our existing tools did not allow for this.

There are a number of different software packages currently available for executing pipelines and building complex workflows, including VisTrails (Callahan et al., 2006), Taverna (Oinn et al., 2006), and Kepler (Ludäscher et al., 2006). Each of these packages provides both a comprehensive underlying framework and a graphical user interface (GUI) for constructing workflows; however, their aim is not to tackle problems specific to neuroimaging and they do not provide the extensive modules and support offered in other packages. This is in direct contrast to Pydpiper: here, the vast majority of our efforts were in constructing modules that are useful for solving neuroimaging registration challenges. The underlying framework, while a robust and necessary part of the toolkit, is not the main focus of Pydpiper.

Several frameworks have been written specifically to address the needs of the neuroimaging community. PSOM (Bellec et al., 2012), written for Octave and Matlab, provides a pipelining overlay to direct scripting level programming, making complicated mathematical and statistical analyses easy to merge with pre-processing. The AIR (Woods 1998a,b) package, written in C, provides source code and examples for running image registrations both within and across subjects and imaging modalities. LONI Pipeline (Dinov et al., 2010) is an extensive pipelining framework that, in addition to its robust underlying architecture, provides an elegant and user-friendly graphical user interface (GUI) for constructing pipelines. Another comprehensive and highly successful neuroimaging toolkit is Nipype (Gorgolewski et al. 2011), a Python-based, open-source software package. Both LONI and Nipype provide interfaces to many common neuorimaging tools such as SPM, FSL, and Freesurfer. These interfaces provide a powerful means for facilitating interactions between these packages. Comprehensive documentation and example scripts are also provided with both, so that users may construct and execute their own workflows.

Although the frameworks described above offer solutions to neuroimaging analysis problems, none of them addressed all of the design principles described in the previous section. For example, while both PSOM and AIR have functionality that overlaps with Pydpiper, PSOM is explicitly intended for developers and if one wants to utilize the source code directly, AIR requires a significant amount of user input and coding in order to execute complex, multi-step registrations^1^. This is in contrast to Pydpiper, which was designed to be accessible to researchers with little coding experience and runs four different types of pipelines upon installation. The GUIs offered by Taverna, VisTrails, Kepler, and LONI mitigate this issue to a degree, though users must still construct their workflows via “box and arrow” graph representations, and with the exception of LONI, were not written explicitly for neuroimaging applications. Even though each framework allows multistage pipelines to be combined into modules, this could still be cumbersome for pipelines with tens of thousands of stages. With Pydpiper, the existing building blocks are structured such that these dependencies are already built into the code, as will be discussed more in the following sections. In addition, because one of our goals was to create a toolkit that would enable non-programmers to write modules, we declined to write a GUI, which, in our experience, tends to dissuade people from exploring the code underneath.

In many ways, Nipype accomplishes much of what we intend to do with Pydpiper, is also written in Python and allows users to write their own code without needing to worry about the underlying architecture. It also provides additional functionality and interfacing that is not included in Pydpiper. As appropriate throughout this manuscript, we provide comparisons between Pydpiper and Nipype. We believe the two toolkits can provide complementary approaches for solving various image processing challenges. In the Discussion section, we outline both scenarios in which Pydpiper might be the preferred toolkit and scenarios where one would prefer Nipype.

Using the aforementioned design principles, Pydpiper was written with four specific applications in mind: (1) iterative, group-wise registration to create a study-specific average; (2) registration of adjacent time points in a chain-like fashion when all subjects cannot be registered together; (3) two-level registration for longitudinal studies where both subject-specific and study-specific averages are created; and (4) an automated multi-atlas label generation procedure. To assist in reusability, Pydpiper provides class types to manage distinct aspects of pipeline creation: “atoms” wrap distinct operations (e.g., registering two images), “modules” link together atoms into reusable processing subunits, and “applications” provide a command-line interface allowing users to drive a particular pipeline. In addition, we created a comprehensive file handling framework to simplify future code development and usage of these atoms and modules. All of this was done with the overarching goal that atoms and modules could be easily combined to create entirely new types of registration pipelines. Moreover, Pydpiper is specifically designed to take advantage of grid computing environments and automatically calculates stage dependencies, decreasing the time necessary for both coding and execution.

In addition to the aforementioned design considerations, we wanted Pydpiper to be a tool that is freely available to the community, with low barriers for adaptation and usage by others. This not only has the effect of continually improving upon Pydpiper, but also increases both transparency and reproducibility of results obtained by using it (Ince et al., 2012). It is distributed under the Modified BSD license, which allows free copying, modification and distribution of the code and is freely available on github (https://github.com/mfriedel/pydpiper). This distributed version control system (git) allows for the tracking of all changes, a complete history of the source code, and the ability to flag issues and discuss them with other developers. As a companion to this paper, a public wiki is also available and contains more detailed information about development, usage and applications. (https://wiki.mouseimaging.ca/display/MICePub/Pydpiper) A virtual machine for code testing and example workflow diagrams are included as well. Additionally, Pydpiper is written in Python and uses the Pyro (https://pypi.python.org/pypi/Pyro4) and NetworkX (http://networkx.github.io/) libraries, all of which are freely available, straightforward to install and enjoy broad support and usage. Pydpiper has been developed for the Linux operating system, the most popular platform currently in use by the neuroimaging community (Hanke and Halchenko, 2011). Finally, we wanted to create a toolkit that could be easily used without extensive programming knowledge. While we welcome and encourage contributions to Pydpiper from expert developers, we structured the classes and example applications such that someone with only a basic knowledge of Linux, Python and Object Oriented Programming could create a pipline specific to their needs.

## 3. Design and implementation

### 3.1. General pipeline and application structure

The core Pydpiper framework that serves as the base for all applications was designed to be as modular and reusable as possible. It is also completely independent of the application being executed. Although we have written this toolkit with an image registration focus, the framework that manages pipeline construction and execution could be used for any type of software engineering paradigm that follows a similar design pattern. This framework is encapsulated in five core classes: *PipelineStage, CmdStage, Pipeline, AbstractApplication*, and *pipelineExecutor*. Taken together, they act in concert to construct pipelines with one or more stages, connect them through a series of interdependencies, execute each stage in the appropriate order via thread pool and encapsulate each pipeline into a larger application that is executed on the command line.

*PipelineStage* is the primary base class upon which all additional executable classes are built. It was designed to contain all of the underlying framework necessary to successfully integrate a single stage into a larger pipeline. This framework includes identifying inputs and outputs, creating and writing to a log file, and keeping track of both stage status (e.g., running, finished, failed) and the amount of memory and processors required for execution. *PipelineStage* also contains the functions that get and set the amount of memory and processors needed for a particular stage as well as those needed for setting the status of a stage (e.g., running, finished, or failed).

The command stage (*CmdStage*) class inherits directly from *PipelineStage*. The primary difference between *CmdStage* and *PipelineStage* is that pipeline stages can run arbitrary pieces of Python code, while command stages are designed to execute individual command line programs. Although our current applications rely heavily on the *CmdStage* functionality, we explicitly wrote *PipelineStage* as the base class, so that Pydpiper users can include pieces of code that don't necessarily require command line execution.

**Figure 2:**
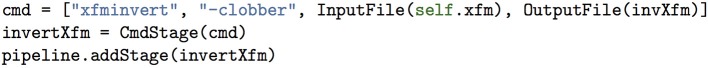
**Example of how to construct an executable Pydpiper stage using the CmdStage class**. The example command used to construct this stage is xfminvert, which takes a transform between two subjects and inverts it. (xfminvert is part of the MINC toolkit, described more fully in section 3.2. A more complete usage example is also provided in section 5). After instantiating the class, it is added to the pipeline via the addStage function. Note that InputFile and OutputFile are themselves classes, designed to indicate to CmdStage the required inputs and outputs for stage interdependencies.

The arguments necessary for running a command, as well as the command itself, are passed to *CmdStage* as an array, appropriately parsed. The command is then executed at the appropriate time using the Python function call. Any command line executable that is called as part of a larger pipeline must be an instance of *CmdStage* and each command stage can run only a single command line executable. Although many command stages are subclassed, as will be described further in section 3.2, they can also be constructed on the fly. If there is a command-line executable that is used only once (and therefore does not warrant its own subclass of *CmdStage*) an array of input and output files can easily be converted to a command stage as shown in Figure 2.

A pipeline (*Pipeline*) is composed of any number of pipeline and/or command stages, and as such, the *Pipeline* class tracks dependencies between stages and keeps a queue of runnable stages and stage state. One of the most critical features of this class is that it infers stage interdependencies based on stage inputs and outputs. That is, if one or more output files from stage A are required for stages B and C, *Pipeline* keeps track of this dependency, and does not add stages B and C to its queue of runnable stages until stage A is complete. Conversely, stages may be executed in any order once all of their dependencies have been satisfied. To capture stage connectivity, the NetworkX library (http://networkx.lanl.gov/) is used to implement Pydpiper pipelines as a directed graph. In addition to the *addStage* command shown as part of Figure 2, *Pipeline* also provides a function called *addPipeline* allowing pipelines to be combined, increasing the ease with which modular code can be written. When stages are added to a pipeline, they are skipped if they already exist. This not only shortens run times, but makes Pydpiper code itself easier to write and read. An example of this type of coding can be found in section 3.2.

In addition to maintaining a queue of runnable stages, *Pipeline* tracks the state of each of its stages (running, finished, or failed). The Pipeline class also uses the Python pickling mechanism, a standard means of object serialization, to save essential pipeline features after each completed stage. This allows an unfinished pipeline to easily be restarted from pickled backup files. The following data is pickled: the directed graph describing stage interdependencies; an array of pipeline stages; the current stage counter; a hash uniquely identifying each stage; a hash of output files for each stage; and an array containing the statuses of each stage. To restart a pipeline, one would simply specify --restart as a command line option when launching pipeline executors, as described below. The --restart option will then load the pickled data into the appropriate variables before starting the pipeline. The graph heads and edges can be quickly reconstructed by iterating through the saved and reloaded directed graph, and all stages with “finished” status are not re-run.

Because of the directed graph architecture of pipelines like this, many stages can be run in parallel, provided their predecessor stages have completed successfully. To run these stages most efficiently, we created the *pipelineExecutor* class. Pipeline executors are managed as a thread pool, with each thread executing individual stages from the pipeline's runnable stages queue. These executors effectively act as clients to the pipeline, which functions as a server. The number of executors required, threads per executor and memory necessary for each process are specified on the command line. Executors can be launched independently, as a stand alone command, or they can be launched as part of an application itself. The values chosen with respect to memory and processors will vary both with an application and available computational resources. Each executor is then initialized as a client of the pipeline server. This client/server architecture is implemented using the Python Remote Objects (PYRO) library (https://pypi.python.org/pypi/Pyro4), and support is included for running on clusters with both the pbs and sge queueing systems. By specifying either --queue=pbs or --queue=sge, Pydpiper will create a script with the appropriate syntax and automatically submit it to the requested queue. For example, by including --queue=pbs –ppn=8 –num-executors =1 –proc=8 –time=18:00:00, Pydpiper will create and submit a pbs script requesting a single node with 8 processors (via --ppn). Once running, this script will launch a single executor with eight threads that will run for a maximum of 18 h.

One of the most salient features of pipeline executors is how they interact with the pipeline. Each executor can consist of one or more threads. In turn, each thread will poll the server to get the next available stage from the pipeline's queue of runnable stages. If enough memory and processors are available to run that stage, the thread will execute the stage. Otherwise, it will sleep for a specified interval before re-polling the server. Once a stage has finished running (or failed to complete), the thread will release the memory and processors used and poll the server again for the next available stage to run. This happens repeatedly by all threads until all stages in the pipeline have finished. Alternatively, if there are failed stages, the pipeline will shut itself down once no more stages can be run. (In this instance, debugging will be necessary before restarting the pipeline). In addition, if an insufficient number of executors were launched, additional executors may be launched at any time via the command line. This may be done whether running locally, or if using an sge or pbs supported cluster.

To tie together command stages, pipelines and pipeline executors into a single runnable program, we created the abstract application (*AbstractApplication*) class. This is the base class for all applications written within the Pydpiper framework. Each class that inherits from *AbstractApplication* will itself be a command line executable that, when launched with the appropriate arguments, will run an entire pipeline from start to finish. This class sets up command line options that are required for all subclasses, initializes the pipeline (or restarts it from backup files) and sets up a logger. It also launches the pipeline daemon, which is where the pipeline is initialized as a server. If the appropriate command line options are specified, subclasses of *AbstractApplication* will launch executors, so that they may begin running immediately. When writing a new application that inherits from *AbstractApplication*, one only needs to extend a few functions without having to worry about the underlying framework. These functions are shown in **Figure 6**. A more complete example of a Pydpiper application that inherits from *AbstractApplication* is included in the section 5.

### 3.2. Class hierarchy and file handling

As noted in the Introduction, Pydpiper supports three main “levels” of classes that are built on top of the core Pydpiper framework described above: atoms, modules and applications. In addition, there is a file handling framework to help simplify their usage. All of the initial classes we developed extend the Pydpiper framework to support files and pipelines that use the Medical Imaging NetCDF (MINC) file format. MINC is a comprehensive medical imaging data format and an associated set of tools and libraries. It was initially developed at the Montreal Neurological Institute (MNI) and is freely available online. (http://www.bic.mni.mcgill.ca/ServicesSoftware/MINC, http://en.wikibooks.org/wiki/MINC). In addition, we make use of pyminc, a Python interface to the MINC2 library (https://github.com/mcvaneede/pyminc). We expect that as development continues (by both us and other members of the community) other file formats will be supported as well.

**Figure 3:**
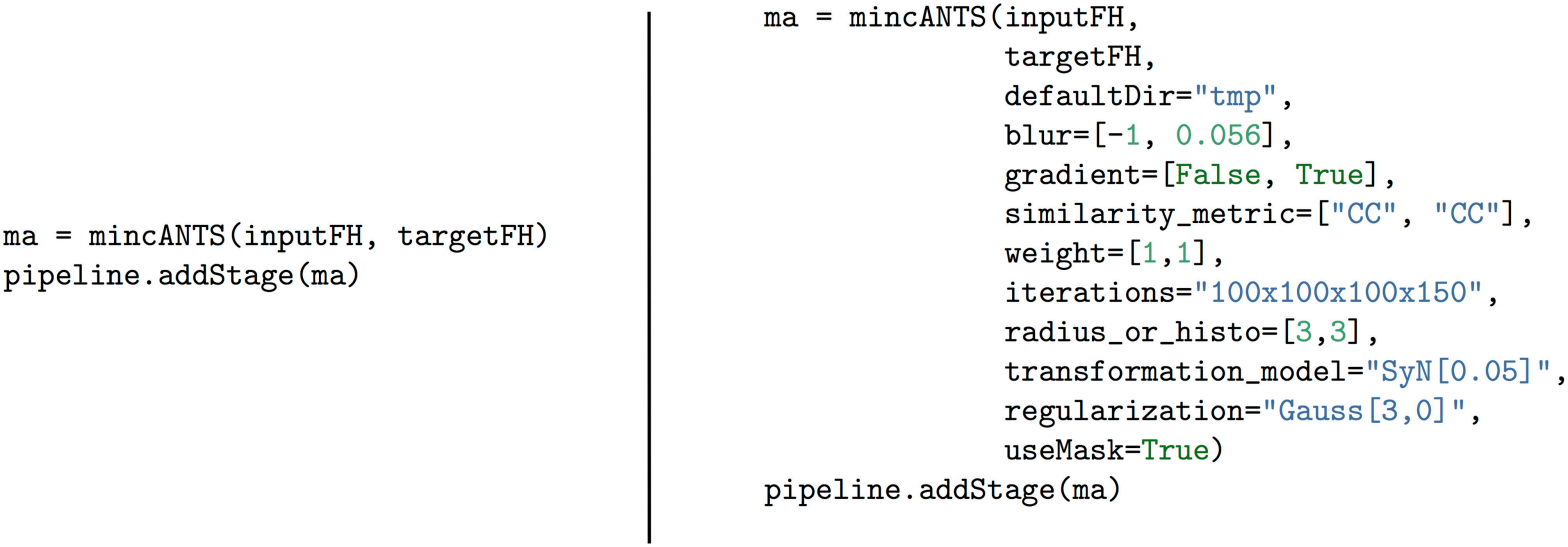
**Simplified call of the *mincANTS* atom (left) and a call that includes all arguments (right)**. The call on the left requires only an input and target file handler, and uses default arguments as *mincANTS* parameters. On the right is a *mincANTS* call that includes specific arguments as parameters, overriding the defaults. These arguments correspond to various command line options required by *mincANTS* and they are discussed in more detail in section 5. We also refer the reader to Avants et al. (2008) and references therein for a complete discussion.

Pydpiper atoms inherit directly from *CmdStage* and act as wrappers around frequently used MINC tools. Each atom has at least one required argument, an input MINC file, which may be passed as a string or a file handler. Additionally, most atoms require a second argument, a target MINC file, which must be passed in the same format (e.g., string or file handler) as the input MINC file. As is noted in the Introduction, image registration determines the transformation mapping one image (source) into the space of another (target), and Pydpiper's atomic structure reflects this. All atoms have multiple optional arguments which are either specified directly or make use of the **kwargs functionality built directly into Python. The choice of optional arguments, and their defaults, were selected based on the most common ways in which we use the MINC tools. An example of minc atom usage is shown in Figure 3. This figure depicts two different ways to call the *mincANTS* atom. This atom calls the command-line program of the same name, the MINC-based implementation of the Advanced Normalization Tools (ANTs) (Avants et al., 2008), a diffeomorphic image registration software package. Whether only two file handlers are specified or the entire list of optional arguments is included, the atom will handle putting together the command to be executed and, because it inherits from *CmdStage*, all of the attributes necesssary to seamlessly integrate it into an existing pipeline are present.

As discussed above, a critical component of running any type of pipeline is keeping track of stage dependencies, inputs and outputs. As is typical of the neuroimaging pipelines that formed the motivation for Pydpiper, each input image in a pipeline is related to others via a series of registrations, transforms and resampling. In addition to stage interdependencies, one also needs to keep track of, for example, the most recent transform between any two images. Or, if a file has been resampled, it may be necessary at a later point to access the original version of the file. Keeping track of these files can be cumbersome, particularly for novice developers, and doing so without resorting to unnecessarily repetitive code can be a challenge. To address this challenge, we have created the *RegistrationPipeFH* class, and its parent class, *RegistrationFHBase*. Each input scan used in a pipeline (typically read in as a command line argument) can be initialized as a file handler (i.e., as an instance of the *RegistrationPipeFH* class). A more complete discussion of how file handlers are instantiated is included in section 5. Although this is not a requirement for using Pydpiper, by using file handlers, all future use of a given input is dramatically simplified. In addition, this class makes it easier to identify the appropriate inputs and outputs to individual stages when constructing new command stages and atoms.

**Figure 4:**
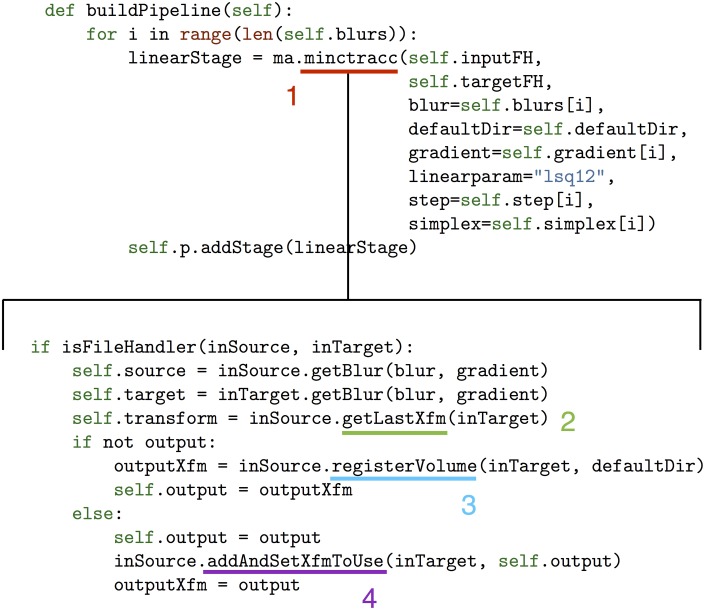
**The buildPipeline function that is part of one of the Pydpiper modules (top) and a portion of the highlighted minctracc class (bottom)**. The minctracc class (1), called multiple times in the for loop, is expanded to show details about how the file handling classes operate. Each time minctracc is called, getLastXfm (2) finds the last transform between input and target and uses it as the input transform for the current function call. If no previous transform exists, an appropriate default is set based on the specified registration parameters. If an output transform is not specified as an argument when minctracc is called (as in this example), registerVolume (3) creates the output file name based on a set of defaults that includes the input and target names and whether or not a previous transform exists between these files. If an output transform is specified, addAndSetXfmToUse (4) adds this transform to the dictionary of transforms between input and target. If the blurs, gradient, step and simplex are not specified when minctracc is called, defaults will be used.

One of the key features of file handlers is the way that they allow access to the state of an image at any stage in the pipeline, and various transforms or resampled files can be retrieved at any time for later use. As a more specific example this, consider the *minctracc* atom, which registers two files based on a specified set of parameters. This atom serves as a wrapper for minctracc, the implementation of the ANIMAL non-linear registration method (Collins et al., 1994, 1995). Although an extensive number of optional minctracc arguments exist, the only requirements for this atom are an input and target. If this input and target are file handlers, minctracc will retrieve the appropriately blurred version of this file (created previously and saved in a dictionary by the file handling class), and set the output transform as the subsequent last transform between input and target, so it can easily be retrieved later if desired. Moreover, if several minctracc calls are made in succession on the same two files, the file handling class will keep track of all previous transforms while still “knowing” which one was the most recent. This results in increasingly simple function calls, particularly within more complex modules. Additionally, any of these transforms can be retrieved at any point in the registration process. An example of this is shown in Figure 4.

**Figure 5:**
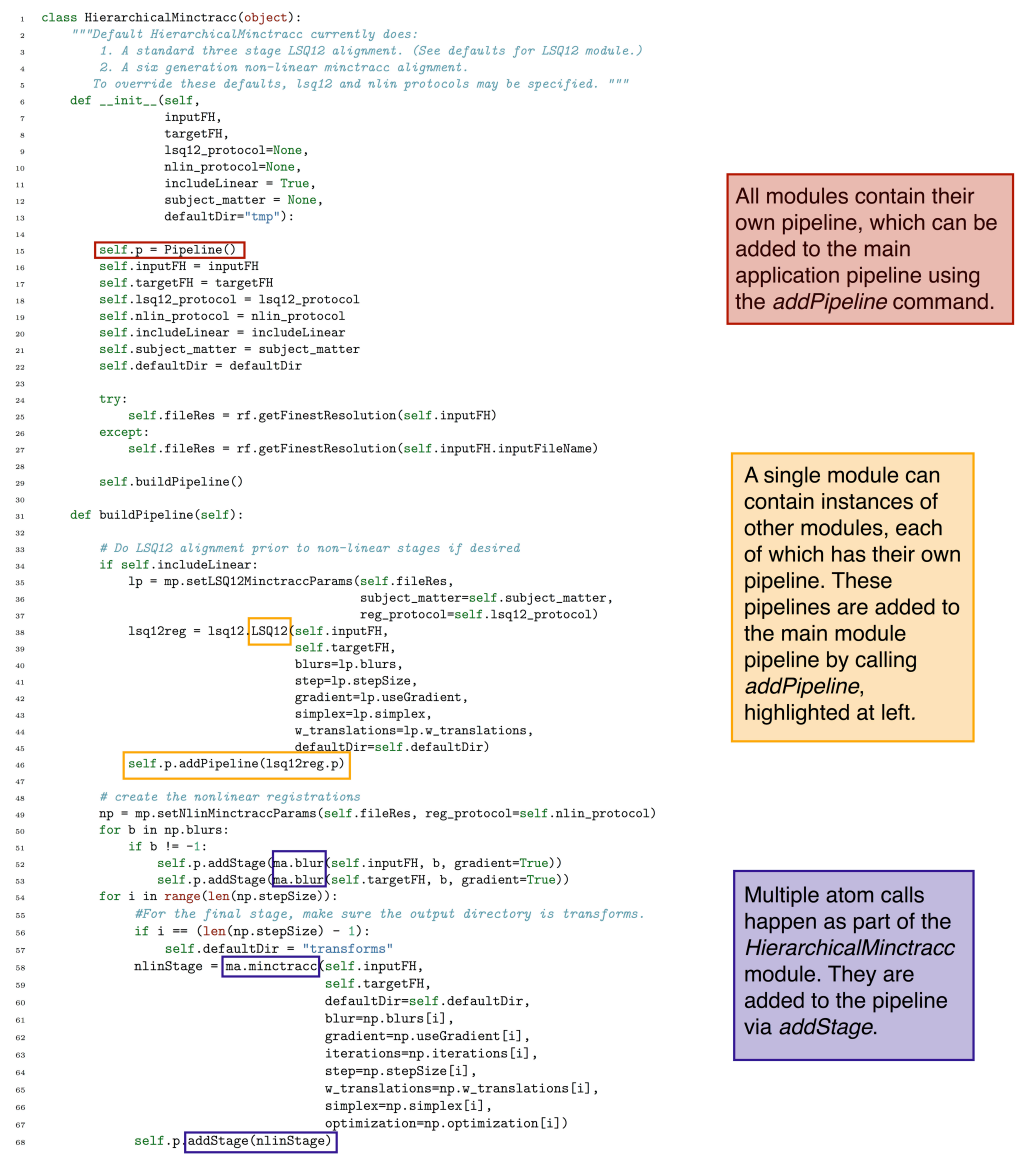
**Code snapshot of the HierarchicalMinctracc class**. In this class, there are calls to both atoms (e.g., blur and minctracc) and modules (LSQ12). Note that minctracc is called iteratively, as is shown in Figure 4, but is using a different subset of arguments.

Modules are perhaps the most flexible and essential component of the Pydpiper toolkit. A module can be composed of a multiple atoms and command stages or a combination of atoms and other modules. Existing modules were designed such that they can be easily pieced together and used in multiple types of pipelines, even for applications that at first glance seem to have quite different architecture. A good example of a Pydpiper module is the *HierarchicalMinctracc* class pictured in Figure 5. This class calls both atoms and other modules and can be easily subclassed or called as is. Including *HierarchicalMinctracc* in a larger pipeline is as simple as instantiating this class as part of a larger module or application (hm = Hierarchical Minctracc(inputFH, targetFH)) and adding it to the existing pipeline (p.addPipeline(hm.p)). Additional arguments (as shown in the __init__ in Figure 5) can be included when the class is called, but are not required.

We noted in section 3.1 that coding with Pydpiper can be done in a non-linear fashion, such that stages in the pipeline are skipped if they already exist. One example of this is depicted in Figure 5. On lines 52–53 of the code, we blur the images associated with inputFH and targetFH. This is done once for each of the blurs specified in the non-linear protocol (self.nlin_protocol), itself defined in the __init__ function. These blurred images are then registered together, by the minctracc call on line 58. (The rationale for blurring is described in more detail in the following section). It is often the case, however, that HierarchicalMinctracc is called in a loop, once for many different input images (each with their own file handler, inputFH) all registered toward the same target (targetFH). Because the same set of blurs is often used, this means that line 53 will construct the exact same pipeline stage multiple times. However, within addStage, there is a check to see if the pipeline already contains an instance of this stage. If it does, the stage is not added again to the pipeline. This results in code that is easy to read (it is conceptually simple to understand why one would want to execute the same command on both an input and target) and write (the programmer does not need to keep track of whether or not the target file has already been blurred in a previous instantiation of HierarchicalMinctracc).

**Figure 6:**
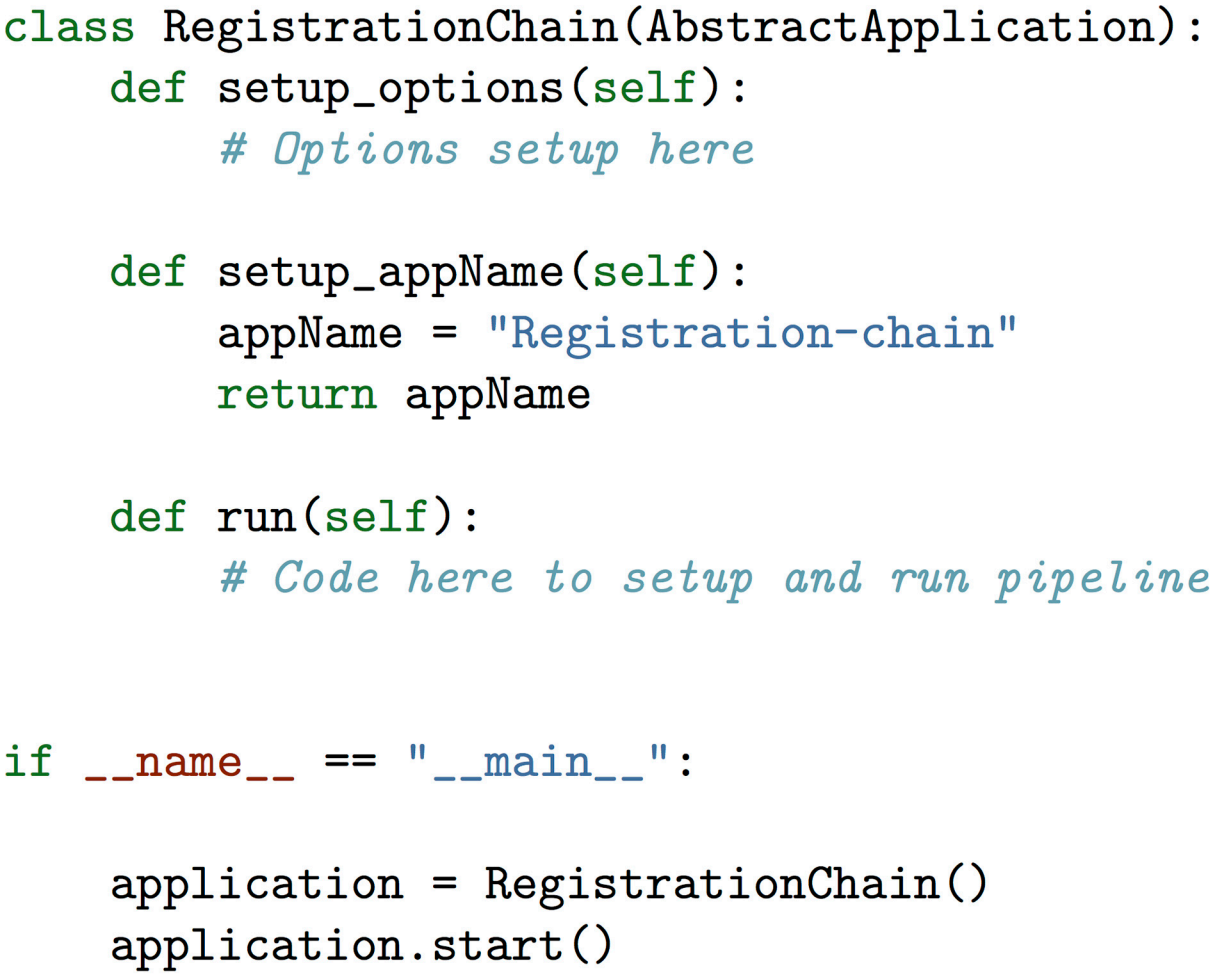
**Example of Pydpiper application code**. Along with the required import statements (omitted from this figure for brevity), the .py file necessary to create an executable for a given application is extremely simple. This example is for the RegistrationChain application described in section 4.3 and is representative of how to construct an application that inherits from AbstractApplication. There are three functions included in RegistrationChain: setup_options, setup_appName, and run. run is the function that calls a unique combination of Pydpiper atoms and modules to construct the appropriate pipeline and in spite of the complexity inherent in this type of registration, this function is less than 100 lines of code. At the end of the file the if __name__ = “__main__” clause is required so that this code can be executed directly from the command line. In section 5, we show a complete example, albeit for a different application, of these functions.

Applications build on both atoms and modules to provide a complete implementation of a single pipeline. The essential feature of an application is that it is a command line executable that inherits from the *AbstractApplication* class described in section 3.1. In theory, an application can be as simple as a single pipeline stage, or one with thousands of stages that are constructed through multiple atoms and modules. Although the complete pipeline for a given application can be extremely complex, at its highest level the application code was designed to be quite simple. This is shown in Figure 6. A more detailed description of each of Pydpiper's current main applications is included in the following section.

## 4. Example applications

In section 1, we briefly introduced the scientific rationale for the applications that motivated the development of Pydpiper. As is noted there, different experimental designs require different registration paradigms. This is particularly true when considering whether and how a common space for all subjects should be created. Nevertheless, commonalities that underlie seemingly disparate registration strategies are largely what shaped the design and development of Pydpiper. In this section, we will describe these common features in more detail and then discuss how they are combined in various ways to address specific image registration challenges.

### 4.1. Essential registration modules

#### 4.1.1. LSQ6

Each input image in a given study is scanned in a slightly different coordinate system, and prior to more precise alignment, it is beneficial if all scans are in the same coordinate system. This happens by applying translations and rotations to each image to align them toward a common target. This common target can be one of the input images, or a specified initial model that is in the desired coordinate system. Because this type of alignment involves six degrees of freedom (three translations and three rotations), we refer to it as LSQ6. For each brain, LSQ6 involves the following steps: (1) blur each input image with a specified Gaussian smoothing kernel (necessary so as not to overly weight singularities or extreme inhomogeneties in an image) (2) align, with a specified registration algorithm, each of the blurred images (3) repeat steps 1 and 2, if desired, for a series of different blurs and (4) resample each input brain with the transform generated from stage 3. The Pydpiper LSQ6 module wraps all of these stages (each of which is its own minc atom) inside a single class. This class takes an array of file handlers (one for each input image in the study) and applies this alignment to each of them.

#### 4.1.2. LSQ12

Whether or not an LSQ6 alignment is required, the next step (or first step) in registering images is often to create an affine alignment between a source and target. This typically involves aligning the source and target via a series of translations, rotations, scales and shears. Because each of these deformations contributes three degrees of freedom, we call this stage of registration LSQ12. Depending on the type of registration pipeline, LSQ12 can be used in different ways. If all subjects in a study are being registered together, it can be beneficial to do an LSQ12 registration between all pairs of subjects in the study (Kovačević et al., 2005) immediately following the LSQ6 alignment. This proceeds similarly to LSQ6: a single LSQ12 call between two brains involves a series of blurs and alignments, with a final resampling of each subject at the end. The goal of this procedure is the creation of an average of all subjects in LSQ12 space. In other types of pipelines, a full pairwise LSQ12 registration is not appropriate due to insufficient homology among subjects, but an LSQ12 alignment between specific sets of subject/template pairs can improve registration accuracy. The Pydpiper LSQ12 module handles both of these instances from a common class.

#### 4.1.3. NLIN

In many ways, the most critical step of image registration is non-linear alignment. This is typically the final stage of image registration, and involves non-uniform deformation of a source image to a target, optimized via a particular metric. In contrast to the LSQ6 and LSQ12 modules previously described, in which all voxels are deformed in a uniform, global way, non-linear registration induces non-uniform deformations. When all scans in a study can be registered together, non-linear registration may happen iteratively, toward an evolving target. After each subject is registered to an initial target (for instance, the LSQ12 average), all subjects are resampled, a new average is created, and alignment proceeds to this new average. Alternatively, a single subject/template pair could be non-linearly aligned with either a single or multi-stage call, but without iterating toward an evolving target. Examples of this include the registration chain paradigm (described in section 4.3) and multiple automated template generation (section 4.5). One of the design goals of Pydpiper was to create a series of non-linear modules that handle either of these registration scenarios in a straightforward way. Moreover, there are multiple different types of non-linear registration metrics that are available (Klein et al., 2009), including Advanced Normalization Tools (ANTs) (Avants et al., 2008) and Automatic non-linear Image Matching and Anatomical Labeling (ANIMAL) (Collins et al., 1994, 1995), the two algorithms we have utilized in Pydpiper. Although they differ significantly “under the hood,” (elastic vs. diffeomorphic optimization, completely different command line options) one of our goals was to implement them such that their usage at a high level is nearly identical. The ANTs toolkit itself provides a number of helpful bash scripts for various types of image alignments, including the type of iterative model building described in section 4.2. However, by incorporating this same paradigm directly into the Pydpiper framework, we have greater flexiblity to use it in conjunction with other Pydpiper modules. In addition, our file handling framework makes it easier to access files created throughout the entire registration process, something that would require additional scripting if using the ANTs toolkit as a stand-alone package.

#### 4.1.4. Pre-processing

In addition to the LSQ6 and LSQ12 modules, there are several pre-processing steps that often need to be included before proceeding with non-linear registration. The most important of these is applying a non-uniformity correction to each image to account for smooth intensity variations that are often present in MR imaging of homogenous tissue (Sled et al., 1998). Another pre-processing stage is intensity normalization, which addresses interslice intensity variations (Zijdenbos et al., 1995). Although each of these steps are most sensibly applied prior to non-linear registration, our goal was to code them such that they could be called at any stage of any type of pipeline. In addition to both of these steps, another step that may be critical to a successful registration is masking. MRI scanning, particularly when done *ex-vivo*, can result in images where a non-negligible amount of tissue is present around the outside of the brain. In order to speed up the registration process and increase its accuracy, a region of interest is defined that encompases the entire brain, and image alignment only occurs within this region. Defining and keeping track of masks and using them when appropriate was also a key feature included in our design and development of Pydpiper, particularly with respect to the file handling class described previously.

#### 4.1.5. Statistics

Finally, the end-goal of performing statistical analysis based on the results of a registration, regardless of type, factored heavily into the design of Pydpiper. For many types of registrations, all statistical analysis must be done from a common space, but how this common space is constructed varies with the type of pipeline. Once a common space has been identified, the full transform from this common space back to each individual subject is used to calculate a deformation field. After smoothing and taking the Jacobian determinant of this deformation field (a measure of the volume expansion or contraction at each voxel) we can use DBM to calculate neuroanatomical differences due to genotype, gender, environmental factors, etc. In particular, the statistics module of Pydpiper was designed with two paradigms in mind: the first was that once the appropriate transform was identified, the calculation of the associated deformation field and Jacobian determinants would proceed as uniformly as possible; the second was that the transform concatenation often necessary to get the appropriate average-to-subject transform would happen in a modular way, independent of determinant calculation, to increase code reusability. This was motivated in part by differences between iterative group-wise registration (section 4.2) and the registration chain (section 4.3). In the latter, deformation fields can be calculated both from a space common to all subjects, or between individual subject pairs, and we wanted code that would handle both in a seamless fashion, particularly at the highest levels.

### 4.2. Iterative group-wise registration

**Figure 7:**
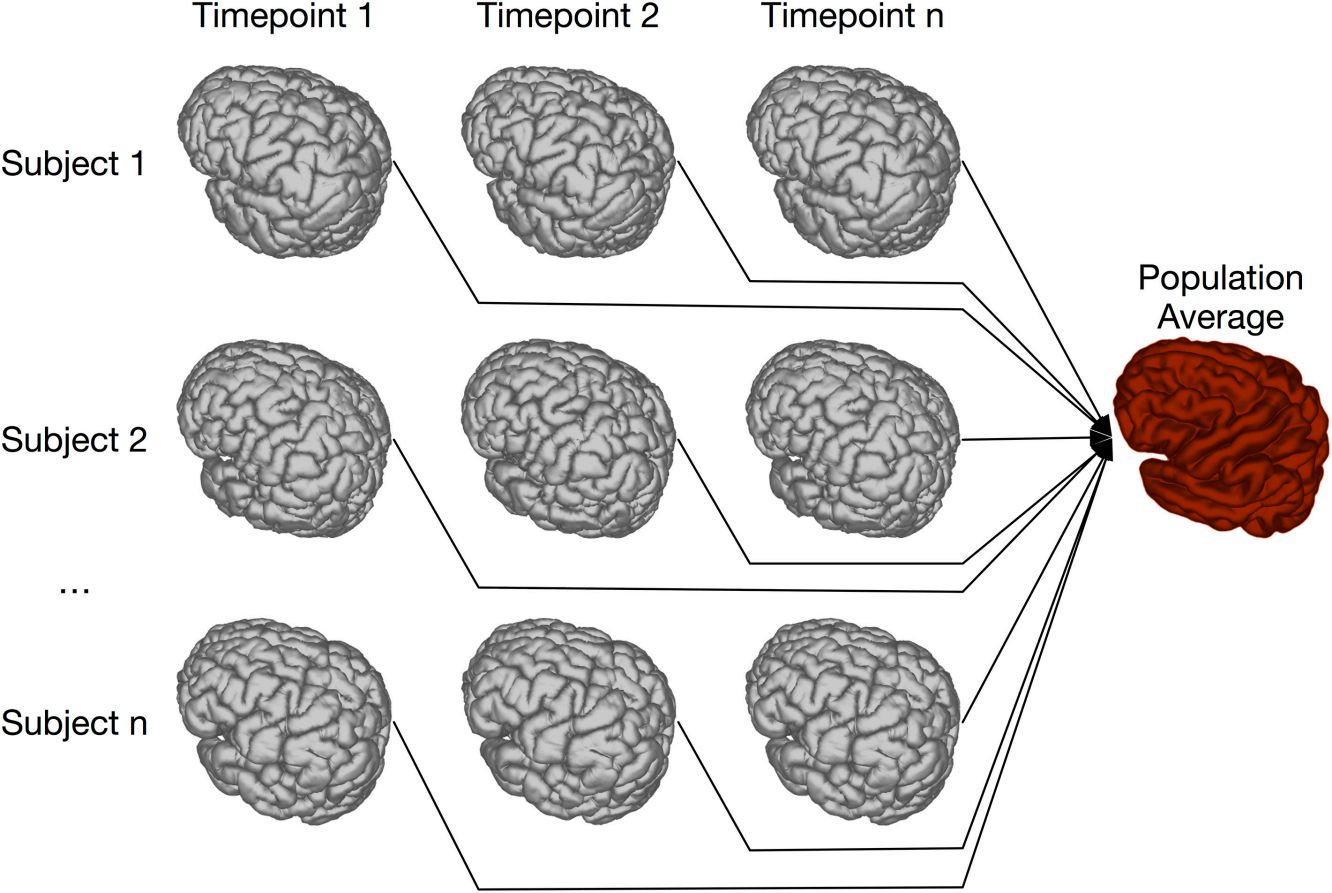
**Schematic of iterative group-wise registration**. This schematic depicts a registration scenario where all subjects, each scanned at one or multiple time points, can be registered to a consensus average.

**Figure 8:**
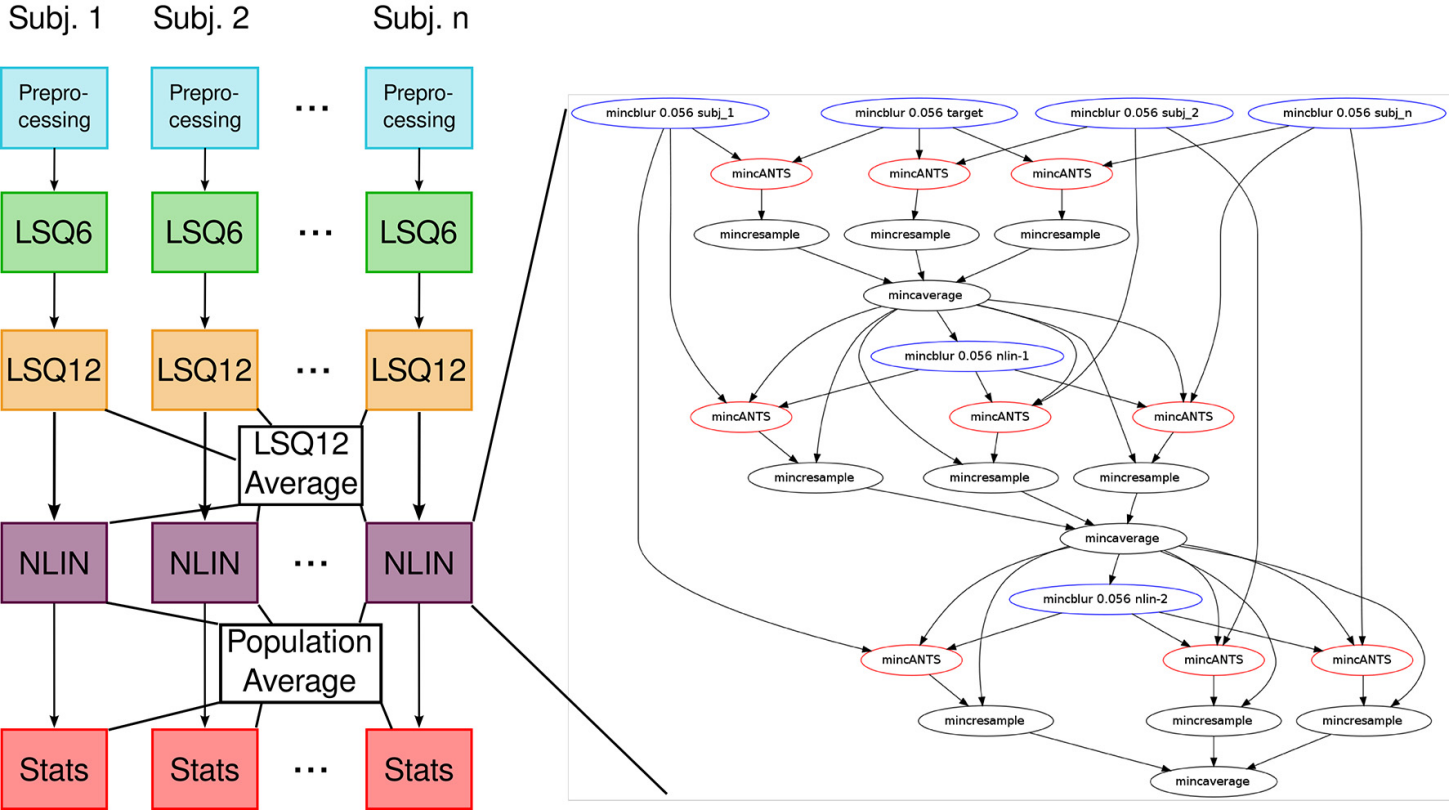
**Code diagram to complement Figures 7, 9**. This figure illustrates the modular nature of how Pydpiper executes each of the stages in this pipeline. Diagramatically, each of the code blocks highlighted in Figure 9 is indicated here as a single unit. One of the non-linear stages is expanded to show the complexity of the pipeline that underlies it.

**Figure 9:**
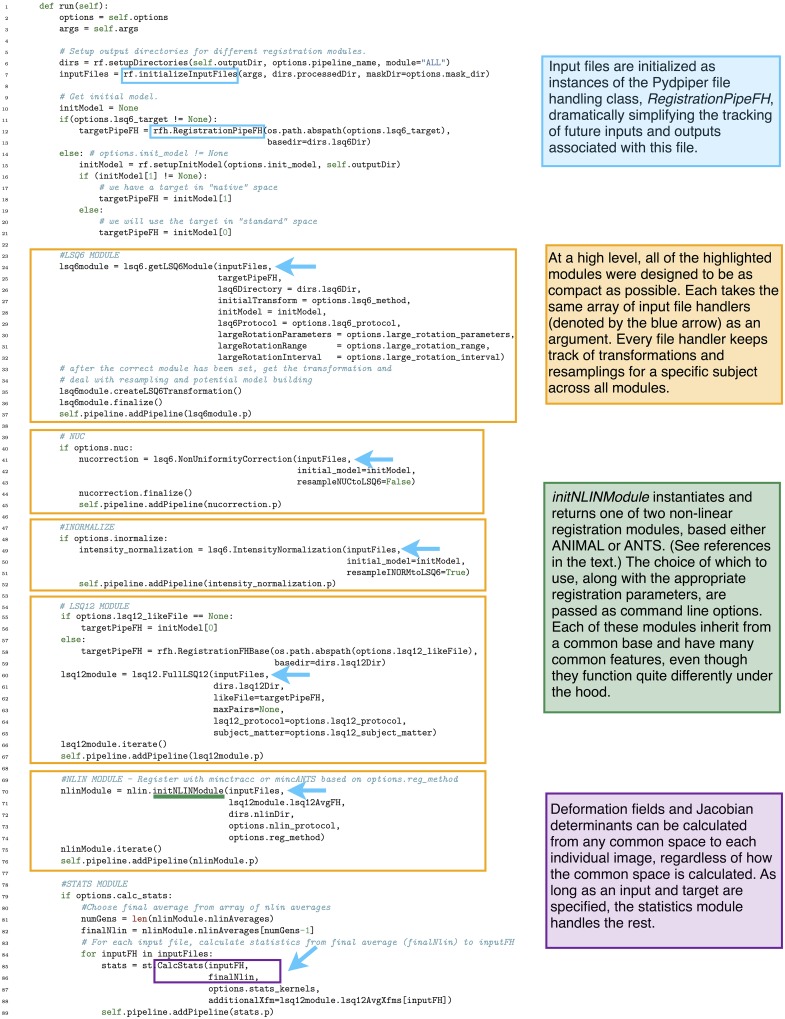
**run() function in the iterative group-wise registration application**. This piece of code illustrates how an extremely complex pipeline can be built up from smaller modules making it simple to read at the application level.

Our previous implementation of iterative group-wise registration is described in more detail in Lerch et al. (2011). In Pydpiper, we utilized the same underlying logic and theoretical framework for this application, but implemented it in a much more streamlined and extensible fashion. Briefly, this iterative, group-wise registration proceeds as follows: we first bring all subjects into a common space using the LSQ6 module. Then, following non-uniformity correction and intensity normalization, we perform a pairwise registration of all subjects in the study using the LSQ12 module. This creates the best possible linear model for this data set. Using the LSQ12 average as a starting template, we then locally deform each scan toward this template, using either an elastic (minctracc, Collins et al., 1994, 1995) or diffeomorphic (mincANTS, Avants et al., 2008) registration algorithm. After this initial alignment, another average is created, and this is used as a template for subsequent non-linear generations. This entire multi-generation procedure is encapsulated in the non-linear (NLIN) registration module. Once a final non-linear average is created, the appropriate transforms are concatenated and used to create deformation fields from this template to each individual subject. These deformation fields are subsequently used in DBM. A schematic of this registration process is depicted in Figure 7. A corresponding code diagram is shown in Figure 8 and the annotated code itself is provided in Figure 9.

One notable feature of our implementation of iterative group-wise registration is that, at a high level, the code is deliberately sparse. The goal of this design was to make each stage (e.g., LSQ6, LSQ12, NLIN, statistics calculations) an independent entity, to aid in both readability and provide a more direct correspondence between the theoretical framework and the code itself. As an example of the size of one of these pipelines, consider an image registration with 10 mutants and 10 wild type mice, the minimum number we typically use for a two group comparison. This pipeline would have a total of 2169 pipeline stages encapsulated into four modules: LSQ6 (including intensity normalization and pre-processing), LSQ12, NLIN and Statistics. For larger studies and the alternate strategies described below, (particularly MAGeT), pipelines can often consist of tens of thousands of stages; however, because of the modular nature of the code, applications remain uncluttered and easy to read.

The modular nature of Pydpiper applications also makes it easier to assess where changes to the pipeline should occur. For example, one might want to proceed directly to non-linear registration after having performed the LSQ6 stage–this could be done quite simply by removing only a few lines of code in the existing application. In addition, each of these modules has a default set of registration parameters that are based on the detected input file resolution. Alternate parameters may be deliniated in a.csv file that is specified on the command line when the application is launched. This makes it simple to flexibly adjust parameters as needed while avoiding hard coded values that are only appropriate for a handful of cases. Another advantage of this modular code is that it is simple to implement alternate registration strategies. For example, the non-linear modules (NLIN) for both minctracc and mincANTS registrations inherit from a common base, which could easily be further subclassed to create an alternate non-linear registration strategy.

### 4.3. Registration chain

There are numerous scenarios where the iterative group-wise registration paradigm described in the preceeding section is inappropriate, and alternative registration and analysis strategies must be employed. This is particularly true in the case of specific types of longitudinal studies, where scans from early time points cannot necessarily be registered to scans at later timepoints, even when doing intra-subject registration. This makes the strategy of registering all brains together in an iterative fashion ineffective. As noted in the Introduction, two examples of this type of study include both tumor growth and normal development. Although it is not possible to register together early and late time points in these types of studies, adjacent time points can often be accurately registered.

**Figure 10:**
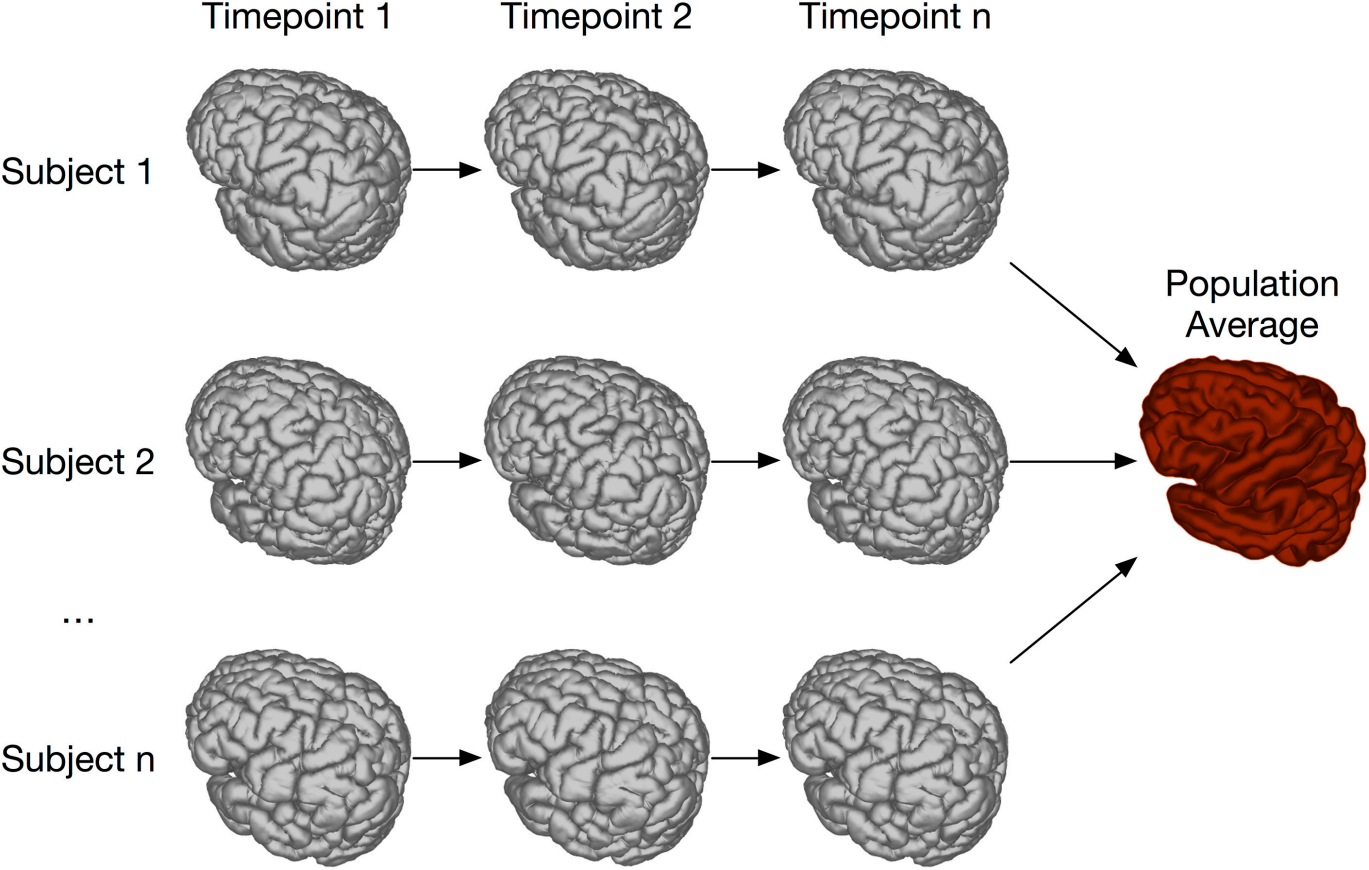
**Schematic of the registration chain pipeline**. In this schematic, each subject is scanned at a series of timepoints. The arrows indicate that, within each subject, timepoint *i* is registered to timepoint *i* + 1. All subject scans at timepoint *n* are then iteratively registered together, creating a common space among all subjects and timepoints. Alternatively, a different timepoint could be chosen as the common space.

In order to address this type of longitudinal study, we have created the registration chain application, schematically depicted in Figure 10. This pipeline works as follows: Each subject is first linearly and then non-linearly registered to the next scan in the time series for that mouse. This is done first through an LSQ12 registration from source (timepoint *i*) to target (timepoint *i* + 1), followed immediately by a non-linear registration from source (*i*) to target (*i* + 1). Once this has been done for all subjects, one time point is chosen as the common time point for the registration. All scans at this timepoint are then registered together via the iterative procedure described previously. This creates the common space required for statistical analysis. The appropriate transforms from this common space to each individual scan are then concatenated and deformation fields calculated.

The code used to accomplish this type of registration has many parallels to the example shown in Figure 9. Like iterative group-wise registration, the registration chain is composed of a number of smaller modules, making the application easy to read. The main registration loop, which aligns scan *i* to *i* + 1 for each subject, is extremely compact: choosing minctracc results in a call to HierarchicalMinctracc, shown in Figure 5, and choosing mincANTS calls a very similar function (LSQ12ANTSNlin), which uses the LSQ12 module in combination with the mincANTS atom to appropriately align input to target. To create a common space for analysis, all subjects at a specified timepoint are then registered together using the iterative procedure described in section 4.2. Deformation fields are calculated from the common space via a subclass of the CalcStats class highlighted in Figure 9.

### 4.4. Two-level registration

**Figure 11:**
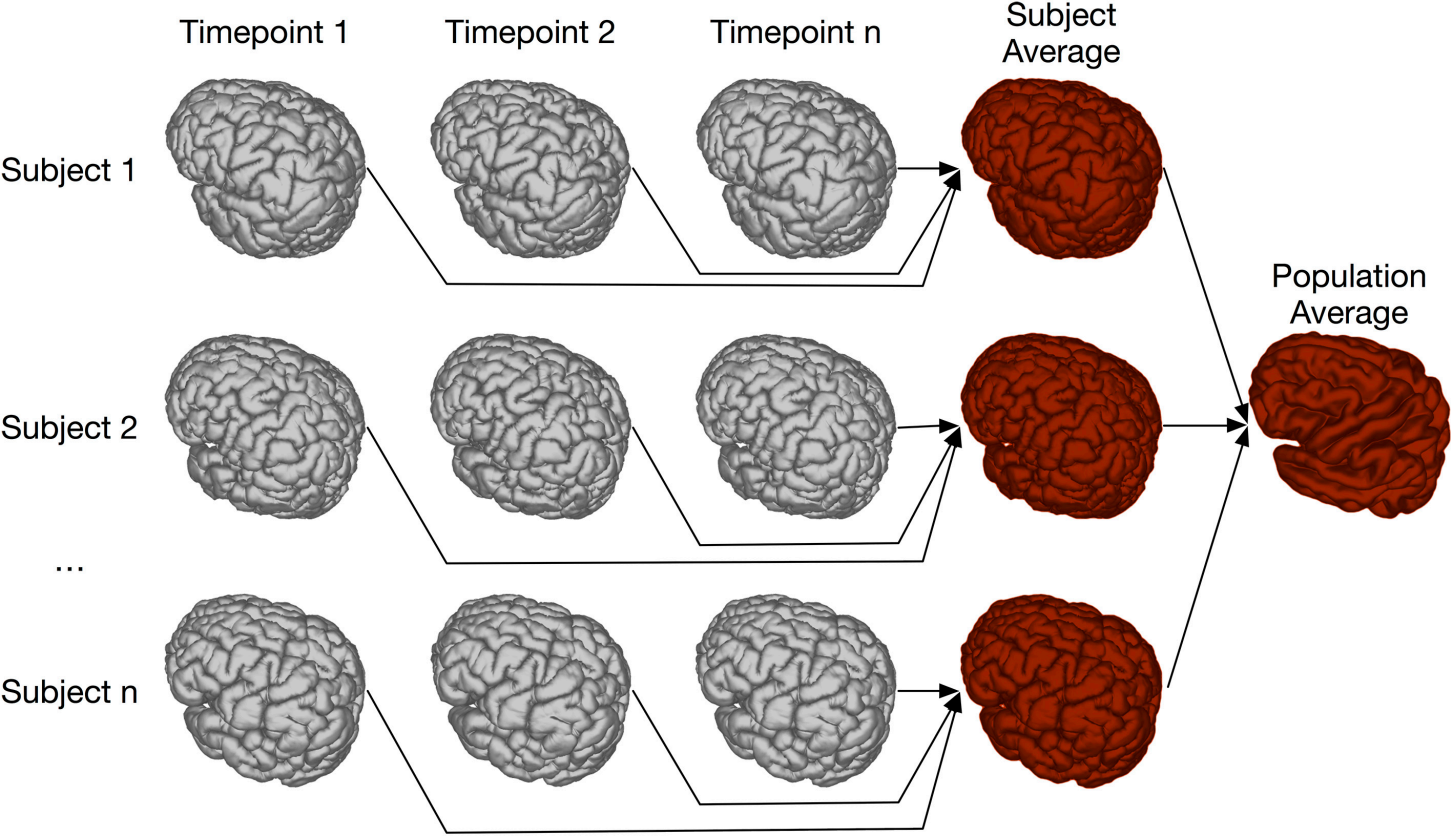
**Schematic of the two-level pipeline**.

Two-level registration is a registration paradigm that creates both subject and population averages. It is appropriate for data sets where all subjects are scanned multiple times, but in contrast to the types of longitudinal registration described in section 4.3, all timepoints for a given subject can be registered together. This is done using iterative group-wise registration to create a subject-specific average, enabling meaningful statistical comparison among all timepoints for a given subject. All of these subject-specific averages are then registered together, again using the iterative group-wise procedure, to create a population average. Transform concatenation can then be used to calculate the appropriate transform from the population average to each subject specific average, and subsequently to each individual scan. This allows for inter-subject comparison at each of the timepoints in the study. A schematic of this is shown in Figure 11.

### 4.5. Multiple automatically generated templates (MAGeT)

Of particular interest in the neuroimaging community is the ability to match MRI volumes to expertly labeled atlases, as structural segmentations are a powerful tool for enhancing analyses (Nieman et al., 2007; Dorr et al., 2008). Unfortunately, creating accurate atlases, particularly across the whole brain, can be challenging. While manual segmentation is often considered the “gold standard” for atlas creation (see e.g., Burk et al., 2004), it is too time-consuming and subjective for the ever-increasing amount of structural MRI data that must be analyzed. As such, automated atlas creation is a powerful and necessary tool and one that we wanted to include in Pydpiper.

**Figure 12:**
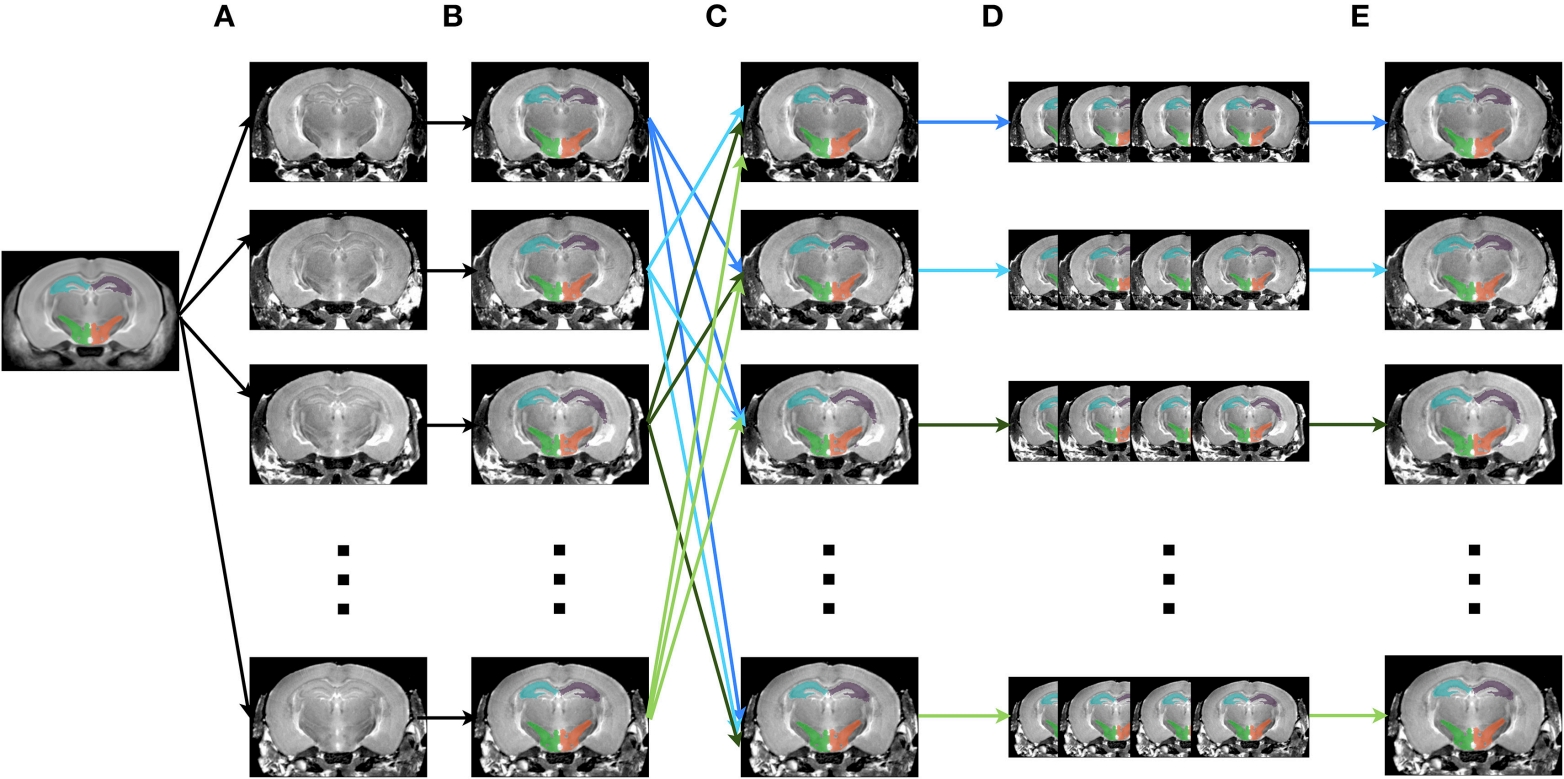
**Schematic of the MAGeT algorithm. (A)** An initial labeled template is non-linearly aligned to a series of subjects. **(B)** Using the transform that results from step A, the labels from the template are propagated to each subject, creating a unique set of labels for that subject. **(C)** Each subject is non-linearly registered to every other subject. **(D)** The initial set of labels from each subject (created in step B) are propagated to every other subject using the transforms from step C. This creates a library of labels for each subject. **(E)** A voxel voting procedure is applied, creating the best set of labels for each subject.

The creation of multiple automatically generated templates from a single labeled brain (MAGeT Brain), as introduced in (Chakravarty et al., 2013), is an example of a multi-atlas based, label fusion technique that produces accurate atlases without the need for manual segmentation. Briefly, it works as follows: using an input template with a set of pre-defined labels, this brain is non-linearly aligned to another subject or set of subjects. Typically, this proceeds first with an LSQ12 alignment, followed by a non-linear registration from source (template) to target (subject). The resulting transforms are then applied to the template labels, such that each subject is now labeled as well. Then, all of the subjects are non-linearly registered together (again, first with an LSQ12 alignment, followed by a non-linear registration), creating a set of labels for each subject. A label voting technique is then applied at each voxel, such that the most frequently occuring label is selected for the final segmentation of that voxel. This whole procedure is graphically depicated in Figure 12. We note that although MAGeT Brain was the explicit motivation for this application, the code could be easily extended to implement more sophisticated label fusion techniques (Wang et al., 2013).

As implemented in the Pydpiper framework, MAGeT re-uses many of the classes and modules from other applications. For example, the alignment of template to subject uses either HierarchicalMinctracc or LSQ12ANTSNlin, exactly as is done for the registration chain. This again illustrates the modular, re-usable nature of this toolkit. Prior to this alignment is the option to use the LSQ6 module for an initial alignment as well. In addition to assessing volumetric differences based on label segmentations, the Pydpiper MAGeT application can also be used in a number of different but related ways. As an example, an input template (or set of templates) can be registered to the population average created from any of the registration pipelines detailed above. After voxel voting (necessary if more than one template atlas is used), these labels from the population average can be back-propagated (via the appropriately concatenated transforms) to each individual subject in the study, enabling volumetric analysis from these sets of labels.

## 5. Annotated code example

In this section, we provide a more complete Pydpiper code example along with a corresponding shell script that one might write to execute some of the same commands. These constrasting pieces of code illustrate the utility of many of the Pydpiper atoms and modules and provide a more detailed example for understanding many aspects of the code discussed throughout this paper. Additionally, because the initial Pydpiper applications are all based on the MINC file format, this section provides a bit more context regarding the command line tools we are using. For more details, we refer the reader to http://www.bic.mni.mcgill.ca/ServicesSoftware/MINC and http://en.wikibooks.org/wiki/MINC.

The example pipeline we show here corresponds to a single iteration of the multi-generation non-linear module discussed in section 4.1, followed by the calculation of the displacement field and Jacobian determinant necessary for DBM. It does the following:

1. Aligns each input subject to a specified template using mincANTS. This will result in a transform from each input to the resulting template. For clarity throughout this section, we will refer to this transform as the “final non-linear transform.”
2. Resample each subject with its unique final non-linear transform.
3. Create an average of these resampled brains to create a new non-linear average.
4. Calculate the linear part of each subject's non-linear transform. The inverse of the full non-linear source-to-target transform is also needed, but is automatically calculated by mincANTS.
5. Concatenate these transforms to calculate the pure non-linear transformation from target to each individual subject.
6. Calculate the pure non-linear vector field for each subject, apply a Gaussian smoothing, and calculate the Jacobian determinant of this smoothed vector field.

Prior to starting this registration, we make the assumption that the input files to this pipeline have already been aligned into a common space by the LSQ6 and/or LSQ12 modules described in section 4.1 of the text.

**Figure 13:**
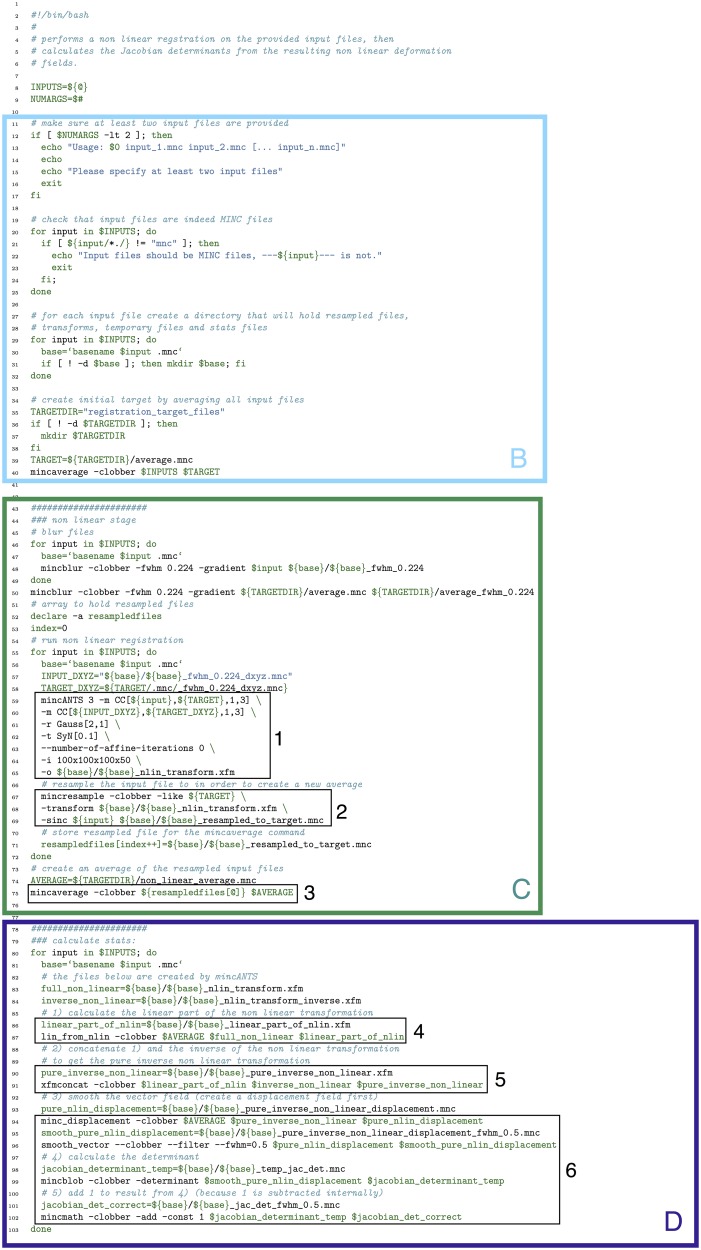
**Bash script that does a non-linear alignment from a set of inputs to a common target, then calculates the resulting deformation fields and their Jacobian determinants**. Note that our labeled sections for this figure begin with section B, as described in the text. **(B)** File checking and initialization of average; **(C)** Image alignment; **(D)** Statistics calculation.

**Figure 14:**
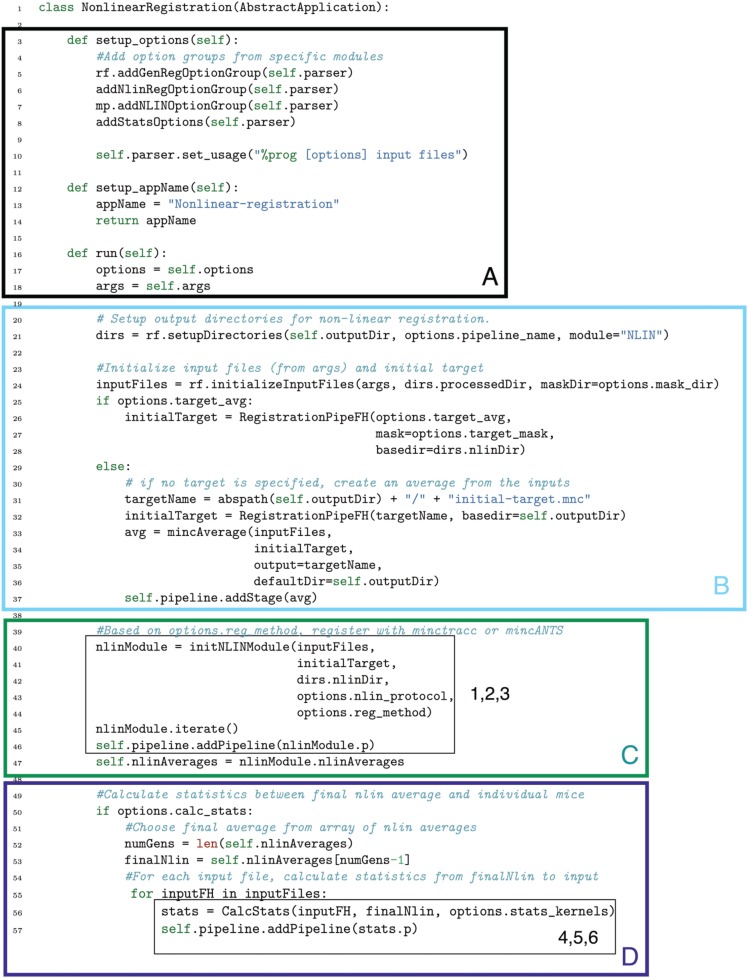
**Non-linearRegistration application in Pydpiper**. This code aligns a set of inputs toward a common target, iterating over multiple generations if requested. Note that we have omitted if __name__ = “__main__” from this figure, but it is included in the .py file that runs this code. (See Figure 6 for more discussion). **(A)** Pre-requisites for AbstractApplication class and integration into pipeline; **(B)** File checking and initialization of average; **(C)** Image alignment; **(D)** Statistics calculation.

In Figure 13 we show how the above pipeline would be executed in a simple bash script. In Figure 14, we show the same pipeline in Pydpiper. In this case, we show the NonlinearRegistration application, which inherits from AbstractApplication and can be run on the command line. Each of these figures has multiple sections of code highlighted, and each highlighted section is labeled. The color and label of one section in Figure 13 corresponds to the same color and label in Figure 14. We will use these labels as a guide for discussion. In addition, registration steps 1–6, as enumerated above, are also labeled in each figure.

### 5.1. Setup and prerequisites

One of the most notable differences between the bash script in Figure 13 and the Pydpiper code in Figure 14 is the initial code set-up and file checking. For the bash script, this is encapsulated in section B, whereas in the Pydpiper code, this is encapsulated in sections A, B. Note that the bash script does not have section A, as it has nothing analogous to Pydpiper's AbstractApplication class.

In section A of Figure 14, there are two functions: setup_options and setup_appName. Both of these are necessary subclasses of AbstractApplication. setup_ options adds various option groups to the application's option parser, to ensure that the appropriate command line options are available. In addition to reducing the amount of hard coding with this application, all of the command line options themselves are grouped together based on their functionality and can be reused in many different applications. setup_appName defines an application name, which is particularly useful for parsing log files. The key thing to note about section A is that, because NonlinearRegistration inherits from AbstractApplication, all of the components necessary for putting together a larger pipeline, calculating stage dependencies, and using the executor model for running multiple stages concurrently is present. No additional setup or coding is needed. In contrast, when using the simple bash script provided, stages can only be run consecutively, one-at-a-time.

In section B of both figures, three things are accomplished, albeit in quite different ways. The first is the checking that is done to ensure that all of the input files are in the MINC file format and that a minimum of two are specified. The second is that output directories are created, one for each input file. Finally, an initial target for non-linear alignment is created by averaging all of the input files.

In Figure 13, file checking is accomplished on lines 12–25 of code. In Figure 14, this happens on line 24 in the function call initializeInputFiles. Not only does this function check for the appropriate number and format of files, but it initializes each of these files as a file handler, as discussed in Section 3.2. In addition to file handler instantiation, if the options.mask_dir argument is specified, a mask will be assigned to each of the input files and their corresponding file handlers. In order to include a mask in the bash script, it would need to be re-written. In spite of the significant additional features this function adds over the corresponding bash script, it contains only 47 lines of code (not shown). Output directory creation happens on lines 29–32 of the bash script, and via two function calls in the Pydpiper code. First, on line 21, the setupDirectories function, used in virtually all other Pydpiper applications to date, creates the main output directories for the registration. Then, as part of initializeInputFiles, a subdirectory is created for each input file.

Finally, on lines 35–40, the bash script calls mincaverage to create an average target from the set of input files. This is accomplished on lines 30–36 of the Pydpiper code, though as is shown on lines 24–28, Pydpiper allows you to specify an initial target on the command line, so averaging is not always necessary. In both Pydpiper scenarios, the target file is initialized as a file handler (lines 26 or 32). Because averaging happens using the mincaverage atom (line 33), all of the appropriate file dependencies are included in the pipeline.

### 5.2. Image alignment

The portion of each piece of code that does image alignment is marked in both figures as section C. In Figure 13, a simple image alignment is shown on lines 46–65. Each input file is first blurred (lines 46–49) with the mincblur tool, using a Gaussian smoothing kernel with a full-width at half maximum (fwhm) of 0.224 μm. The target is blurred as well (line 53). Then, the blurred version of each input file is aligned to the blurred version of the target via a mincANTS call (lines 59–65). This particular call uses a cross-correlation similarity metric (CC) with a Gaussian regularizer (Gauss[2,1]) and a transformation model that uses symmetric normalization (SyN[0.1]). More details about these parameters can be found in Avants et al. (2008). The resulting transform is then applied to each of the input subjects via a mincresample call (lines 67–69) and a new average is created via mincaverage (line 75). Although this is a straightforward and brief script, it requires editing for any set of images that do not use these hard coded parameters, and extending it to multiple generations would require a fair amount of recoding.

The Pydpiper code that accomplishes this same alignment is effectively encapsulated two function calls, shown on lines 40–45 of Figure 14. First, the initNLINModule function is called on line 40. This function returns the appropriate non-linear module as nlinModule. The module returned depends on the value of options.reg_method passed into the function. In the example here, options.reg_method=mincANTS is specified on the command line, and initNLINModule returns an instance of NLINANTS.

After the instantiation of NLINANTS, the iterate() function is called. This function executes the following commands: After blurring both input and target using the blur atom, the blurred version of each input is registered to the blurred version of the target using the mincANTS atom. Then, as in the bash script, the resulting transform is applied to each input, and it is resampled via the mincresample atom. Then, the mincaverage atom is used to create a new non-linear average. (If additional generations were required, the new average would be blurred, and each blurred input would be registered to this new average, with the entire cycle repeating). Note that each of these atoms calls the command line tool of the same name, and the commands exectued are nearly identical (provided the same set of parameters) as those shown in the bash script.

The exact registration parameters used by NLINANTS, including (but not limited to) the Gaussian smoothing kernel necessary for blurring, the similarity metric for alignment and the transformation metric are all contained in the file specified for options.nlin_protocol (line 43). If no protocol is specified, a set of defaults, currently optimized for registration of mouse brains, is used. For the present example, the parameters necessary for only one generation are included in the protocol file. In contrast to the bash script, simply updating the non-linear protocol extends the code to an arbitrary number of generations. No re-coding is necessary.

### 5.3. Statistics calculation

Finally, in section D of each figure, we show the code necessary for performing a statistics calculation. As is evident from the bash script in Figure 13, calculating a Jacobian determinant is a multi-step process: First, the linear part of the non-linear transform from input to target is calculated (line 87). Then, this transform is concatenated with the full transform from target to input (automatically calculated by mincANTS during the alignment procedure) via xfmconcat on line 91. After a calculation (line 94) and smoothing (line 96) of the displacement field, the Jacobian determinant is calculated (lines 99–102). Note that the determinant smoothing happens for only a single blurring kernel (in this case, the specified fwhm is 0.5 μm), and keeping track of all the inputs and outputs is a critical step in making sure this script executes properly.

In constrast, the Pydpiper execution of this code is contained entirely on line 60. For each input and target, the CalcStats class is instantiated. Within this class, fullStatsCalc executes each of the same stages as in the bash script using the appropriate atoms and modules. The deformation field may be smoothed with more than one blurring kernel (a list is specified as the --stats-kernels command line option). This list of blurs is passed as the options.stats_kernels argument to CalcStats and results in the calculation of multiple Jacobian determinant fields. Additionally, on lines 52–53, the target file necessary for the statistical calculations is selected as the final average from a series that may be generated; in the current example, this number is one, but will be larger for multi-generation registration.

Finally, we note the similarities between Figure 14 and Figure 9. In particular, the code in sections C, D is nearly identical to that on lines 69–89 of Figure 9. This module reusibility was a deliberate design choice.

### 5.4. Running the code

To run the bash script depicted in Figure 13, assuming it is located in an appropriate directory in the user's path, the command is:


nlin_registration_and_stats.sh input_1.mnc
     input_2.mnc ... input_n.mnc


The analagous command for the Pydpiper code is:


NLIN.py input_1.mnc input_2.mnc ...
     input_n.mnc –calc-stats
--nlin-protocol=ANTS_protocol.csv
     --mask-dir=/directory/of/masks
--num-executors=1 –proc=8


The command line arguments for both the bash script and Pydpiper code are simply the brains to be registered (input_1.mnc ... input_n.mnc). Additional command line options are also specified for the Pydpiper code. --calc-stats is required for the final statistics calculation. (If this option is unspecified, the non-linear alignment will run but no statistics are calculated). --nlin-protocol supplies a non-linear protocol for registration, and --mask-dir specifies a directory of masks to be associated with each input. Additionally, the --num-executors and --proc options are not required, but if they are unspecified, the NLIN.py command will launch the pipeline server only, and executors will need to be launched separately.

## 6. Discussion

The ability to use neuroimaging technologies to help understand the relationship between genotype and phenotype will be an important contribution to biomedical research in the twenty-first century. Although there are multiple different methods for analyzing neuroimaging data, image registration is of particular interest due to its wide range of applications. Performing image registration in an accurate and automated way is a critical component of of many neuroimaging studies, regardless of subject-type (humans, mice) or imaging modality (MRI, micro-CT, OPT). Different experimental designs require different registration strategies in order to assess growth patterns, compare genotype differences, or look at the impact of learning. Nevertheless, common features underlie these registration strategies, suggesting that a common computational framework may be used to construct a multitude of different registration pipelines. With the Pydpiper toolkit, we have created such a framework.

Throughout this paper, we have discussed many of the design choices that influenced our development of Pydpiper. Above all else, we were motivated by five principles: (1) high-level coding should be as simple as possible for those with less coding experience (advanced users can still easily get “under-the-hood” to create new modules); (2) individual building blocks of code should be as modular as possible, easy to subclass, and geared toward a range of biologically relevant applications; (3) complete, runnable pipelines containing thousands of stages and addressing the registration scenarios described above should be available “out-of-the-box”; (4) at the end of any pipeline, there should be an option to calculate the derived volumes necessary for TBM based statistics, using a module that contains all of the required stages; (5) we should include a robust file handling class to keep track of naming schemes and file interactions across many modules in a single application. Stemming from these principles, we believe that Pydpiper offers the following innovations to the community:

- A robust file handling class that allows access to outputs from all stages of registration at any point in the pipeline. To the best of our knowledge, no other package offers a similar framework.
- The ability to write code in a “non-linear” way; that is (as shown in Figure 5), duplicate stages that make conceptual sense can be written into the code, but are only executed once. This results in code that is both easy to read and write.
- A set of classes (in the form of atoms and modules) that are reusable, easy to subclass and designed to be combined in different ways to solve a variety of image registration problems.
- A toolkit that enables novice programmers to quickly piece together relatively complex pipelines with only a few lines of code.
- Four complete applications that run complex image registration pipelines with thousands of stages, “out-of-the-box.”

As we noted in the Introduction and throughout the text, there are a number of pipelining frameworks currently available for running image registrations, and although our goal is not to replace any of them, we believe we offer complementary functionality. This is particularly true for Nipype, which is also open-source, written in Python, and has many of the same goals as Pydpiper. At present, Nipype offers interfaces to many more common neuroimaging toolkits than Pydpiper, and if one wanted to create a pipeline using any of these tools (e.g., FSL, Freesurfer, SPM), Nipype is the obvious choice. For other applications, such as an iterative registration using ANTs, one could choose either framework, as both Nipype and Pydpiper provide the infrastructure to do this relatively easily. Where we believe Pydpiper offers an advantage is via the integration of the file handling class into the high-level code structure. Our toolkit gives users the ability to quickly put together applications from our existing modules with relatively simple syntax, and through the file handlers, have the ability to access the state of each input at any stage throughout the pipeline. In particular, using the file handling framework in conjunction with the statistics module gives users a significant amount of flexibility in calculating statistics, making it easy to perform TBM at the end of any pipeline.

We hope that our architectural goals and code construction will attract both seasoned developers and more novice coders who want to tackle a variety of registration challenges, without having to piece together a mish-mash of functions from scratch. By creating Pydpiper as an open source, freely available toolkit, we also hope to facilitate significant additional contributions from the community. With the emergence of new imaging techniques and experimental designs will come the need for new registration paradigms, and we expect that the existing Pydpiper code provides a solid foundation on which to build these new pipelines.

### Conflict of interest statement

The authors declare that the research was conducted in the absence of any commercial or financial relationships that could be construed as a potential conflict of interest.

## Footnotes

^1^We note there that AIR is a module that can be used within LONI. In this instance, we are talking about compiling and using the source.
